# Anthropogenic and environmental factors associated with koala deaths due to dog attacks and vehicle collisions in South-East Queensland, Australia, 2009–2013

**DOI:** 10.1038/s41598-023-40827-w

**Published:** 2023-08-31

**Authors:** Ravi Bandara Dissanayake, Mark Stevenson, Viviana Gonzalez Astudillo, Rachel Allavena, Joerg Henning

**Affiliations:** 1 School of Veterinary Science, The University of Queensland, Gatton, QLD 4343 Australia; 2 Melbourne Veterinary School, The University of Melbourne, Parkville, VIC 3010 Australia; 3 Present Address: Food and Agriculture Organisation of the United Nations, Rome, Italy

**Keywords:** Ecology, Ecological modelling, Risk factors

## Abstract

Populations of the iconic Australian koala are under constant decline. Their deaths are associated with rapid and extensive urbanization and the fragmentation of habitat areas. Using citizen science data on reported koala mortalities, we quantified the association between anthropogenic and environmental factors and the two leading causes of koala deaths, dog attacks and vehicle collisions. We achieved this objective using a case–control study design to compare the odds of exposure to a given risk factor for cases (a given cause of death) with the odds of exposure to a given risk factor for controls (all other causes of death). Koala deaths due to dog attacks were positively associated with registered dog population density and negatively associated with lot density whereas koala deaths due to vehicle collisions were positively associated with road density (road length per square kilometer) and negatively associated with human population density and distance to primary and secondary roads. The results of this research can be used to develop strategies to mitigate the risk of deaths due to dog attacks, for example by conducting educational awareness programs, promoting registration of dogs and discouraging free roaming of dogs. In a similar manner, in high-risk areas for vehicle collisions, over- or underpasses can be built to facilitate safe movement of koalas for road crossings or speed limits could be introduced to reduce the likelihood of premature koala deaths due to vehicle collisions.

## Introduction

Conservation of endangered and declining wildlife is becoming increasing challenging due to constantly changing and growing threats, locally and globally^1^. Habitat loss, climate change, predation through introduced species and deaths due to (direct or indirect) trauma are major threats to wildlife worldwide, while illegal trapping and poaching play a significant role in some countries^2^.

In Australia, populations of the iconic koala (*Phascolarctos cinereus*) are under constant pressure through a variety of ecological and anthropogenic drivers^3^. Reasons for the decline in koala numbers in Australia have evolved over time^3–6^ with hunting having a major impact on populations in the nineteenth century^3,7^ compared with degradation and fragmentation of habitat, rapid and extensive urbanization and natural disasters such as floods and bushfires impacting koala populations in the early twenty-first century^6–8^. On 12 February 2022 the koala was reclassified as endangered under the Environment Protection and Biodiversity Conservation Act 1999, after previously being listed as vulnerable since 1999^9^.

Challenges in implementing mitigation strategies to prevent premature koala deaths arise from unsuccessful or insufficient efforts by local authorities to stem habitat clearing, a limited understanding of risk factors associated with koala deaths and a lack of robust information on mortality risks and on where koala mortalities occur^10^. A number of studies have assessed preventable causes of deaths in koalas^11–13^. While providing a useful starting point to guide development of more pro-active approaches to manage preventable mortality risk, a criticism is that these studies have focused on relatively small study areas^14^ and, as a result, have failed to document risk factors consistent across larger geographical scales.

In the Australian jurisdictional state of Queensland, the greatest concentrations of koalas are found in South-East Queensland (SEQLD)^15^, but since the 1990s, koala populations have declined in this area due to various reasons^16^. A longitudinal analysis conducted using wildlife hospital records identified injuries caused by dog attacks, vehicle collisions, infectious diseases such as chlamydiosis and wasting syndrome as the most common causes of death among koalas in SEQLD^11,12^. This research also identified spatial and temporal “hotspots” of koala deaths in SEQLD which are in need of further investigation^12^. To improve conservation outcomes for SEQLD koalas, identifying and quantifying the association between anthropogenic and environmental factors and cause-specific koala mortality risks at the regional level is required^14^.

The aim of this study was to quantify the association between anthropogenic and environmental factors and dog attack and vehicle collision koala mortalities across SEQLD. We achieved this objective using data collected from a wildlife hospital and using a case–control study design approach to compare the odds of exposure to a given risk factor for cases (a given cause of death) with the odds of exposure to a given risk factor for controls (all other causes of death). A secondary aim was to provide, based on our research outcomes, guidance on how citizen science data might be used to more rigorously inform koala conservation policies.

## Materials and methods

### Study area

**Figure 1:**
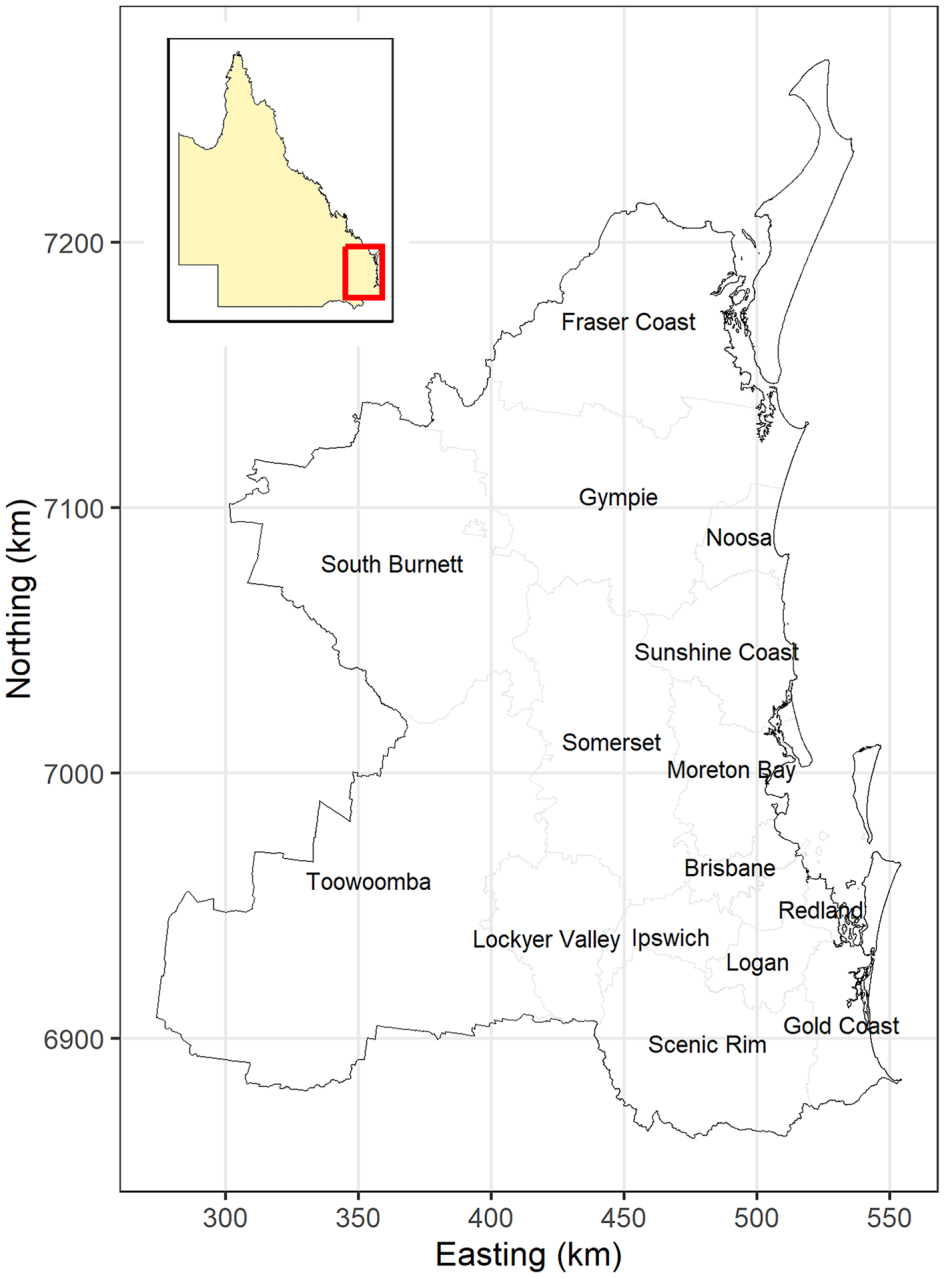
Map showing the boundaries of local government areas in South-East Queensland, Australia, where the study of risk factors associated with koala deaths due to dog attacks and vehicle collisions was conducted. The map boundaries for this (and subsequent maps in this paper) were obtained from the Australian Bureau of Statistics (URL: https://www.abs.gov.au/). This map (and subsequent maps in this paper) were created using R using the contributed sf, raster and ggplot2 packages.

The area for this study comprised 15 Local Government Areas (LGA) in SEQLD (each administered by a local government or council), inclusive of 1448 Australian Bureau of Statistics suburb and local areas (SALs) covering an area of 57,623 square kilometers (Fig. 1). The eastern part of the study area, collectively known as the Koala Coast (including portions of the LGAs of Redland, Logan and Brisbane) and Pine Rivers (portion of the Moreton Bay LGA) is regarded as prime koala habitat that has a relatively high human population density with rapid urban development in recent years^17^. The western part of the study area also contains habitat suitable for koalas but has a lower human population density, compared with the east.

### Koala mortality data

Data on koala submissions to a central wildlife hospital within the study area were retrieved from KoalaBASE, an online database developed and hosted by the University of Queensland since 2015 and owned and managed by the Queensland Department of Environment and Science^18^. KoalaBASE records demographic and clinical case information on koalas submitted to wildlife hospitals in SEQLD from 1997 onwards and incidental koala sighting details from 1997 to 2014.

The data used for these analyses were for the 5-year period from 1 January 2009 to 31 December 2013 and included reports for the three most common causes of koala mortality: dog attacks, vehicle collisions and chlamydia-like signs^12^. This time frame was selected to allow the location and timing of recorded koala deaths to coincide with the available mortality risk factor data, as explained in detail below.

Details for each koala that was either found dead or needed to be euthanised after submission to the wildlife hospital included the longitude and latitude coordinate of the site where the koala was found as well as the name of the LGA or SAL in which the koala was found. Only those koala deaths with recorded location details were used to develop the statistical models (Table 1).

**Table 1:** Number of koalas deaths (by cause of death) reported by members of the public between 1 January 2009 and 31 December 2013 in South-East Queensland, Australia. The data were retrieved from KoalaBASE (URL: https://www.koalabase.com.au).

Cause of death	Number of koalas with location details		Total
	Present	Absent	
Dog attack	395	0	395
Vehicle collision	1431	106	1537
Chlamydia-like signs	943	2	945
Total	2796	108	2877

### Explanatory variables

Environmental and anthropogenic risk factors (referred to as explanatory variables) hypothesised to be associated with koala mortality included human population density, height above sea level (in meters), distance to primary and secondary roads (in kilometers), road density, dog population density, indices of remoteness and lot density. Lot density was defined as the number of contiguous “lots” of land per square kilometer where, for the most part, a lot was assumed to be a tract of land under single ownership.

The selection of explanatory variables in this study was guided by existing literature.

Human population density is directly linked to greater urbanization and thereby increases in traffic volume^19,20^, but also an indicator for pet ownership^21^. Median elevation above sea level was selected as elevation influences koala habitat type^22,23^. Both explanatory variables were considered in the models to identify risk factors for mortalities due to dog attacks and vehicle collisions.

Road density is a typical characteristic of urban sprawl^20^, while distance to primary and secondary roads is an indicator for human access to koala habitat^24,25^, but also for habitat fragmentation which increases the likelihood of vehicle collisions. Remoteness was considered to represent the larger activity range of free-roaming dogs in less populated areas^26^. Lot density represented a proxy for the number of dogs that might be kept on a property-for example, some LGAs provide guidance on the number dogs that are permitted to be kept, dependent on property size^27^.

Our methodology for developing raster maps of the explanatory variables is as follows. Human population counts expressed as the number of individuals per mesh block were retrieved from the Australian Bureau of Statistics Census of Population and Dwellings 2011^28^. Mesh blocks are the smallest administrative unit defined by the Australian Bureau of Statistics and represent areas that contain, on average, between 30 and 60 dwellings. Population counts were assigned to the centroid of each mesh block within the study area ($n$= 42,791) and a 1200-m bandwidth, 500 row by 500 column raster map developed using kernel smoothing^29^. Each cell of the raster map represented the number of individuals present (as of census night, 2011) which was then converted to human population density, expressed as the number of individuals (× 1000) per square kilometer.

Elevation data for the study area, expressed as height (in meters) above sea level at a resolution of 3 s (approximately 30 m) were retrieved from the Queensland Spatial Catalogue, QSpatial^30^. Processing of the elevation data involved interpolation to return a 500 × 500 cell raster map showing median elevation for each raster cell.

Locations of primary and secondary roads in SEQLD, as of December 2011, were obtained from Openstreetmap^31^. Using the contributed spatstat package^32^ in R^33^ a raster map was developed showing, for each cell, the distance in kilometers to the nearest primary road. A similar approach was taken for secondary roads. Road density was calculated as the length (in kilometers) of primary and secondary roads per square kilometer. Remoteness indices (expressed as the time taken, in hours, to reach the nearest city of more than 50,000 inhabitants) and lot density (the number of land parcels per square kilometer) were retrieved from the Queensland Spatial Catalogue^30^. The distance to primary road, distance to secondary road, remoteness and lot density raster maps were interpolated to return maps of the same dimensions as those for human population density and elevation.

To develop a raster map of registered dog density, dog control officers from each of the 15 LGAs included in the study area were contacted either by email or phone and asked to provide counts of dogs registered within each SAL. Of the 15 LGAs, 14 provided dog counts at the SAL level and one provided data at the LGA level (Supplementary Table S1).

SAL human population counts were retrieved from the Australian Bureau of Statistics Census of Population and Dwellings 2011^28^. These and individual mesh block human population counts were used to determine the proportion of a SAL’s (human) population that was resident in each mesh block. These proportions were then applied to the SAL registered dog population counts to return the estimated number of registered dogs per mesh block.

For the single LGA that provided registered dog counts aggregated to the LGA level, the proportion of the LGA’s human population residents in each mesh block was calculated. These proportions were applied to the LGA registered dog population count to return the estimated number of registered dogs per mesh block for that LGA.

Registered dog population counts were assigned to the centroid of each mesh block and a 1200-m bandwidth, 500 row by 500 column raster map was developed using kernel smoothing, similar to the approach used to estimate human population density. This returned the estimated number of registered dogs present in each raster cell which was then converted to registered dog population density, expressed as the number of registered dogs (× 10) per square kilometer.

Digital maps of SEQLD LGAs, SALs and mesh block boundaries (projected in the Geocentric Datum of Australia 2020 [GDA2020] EPSG code 7844) were retrieved from the Australian Bureau of Statistics (URL: https://www.abs.gov.au/). For mapping, the Australian Bureau of Statistics maps were reprojected to WGS 84 UTM zone 56S (EPSG code 32756). Our maps were created using R using the contributed sf^34,35^, raster^36^ and ggplot2^37^ packages.

The anthropogenic and environmental risk factors considered for the dog attack and vehicle collision models were tested for bivariate collinearity using the Pearson’s correlation coefficient^38^. If the correlation between a pair of risk factors was greater than 0.5, the more biologically plausible of the two was selected for further analysis.

### Case–control study

For our case–control study analyses koala deaths for a given cause of mortality were selected as cases and koala deaths for all other causes were the controls. This meant, for example, that for the analysis in which the objective was to identify risk factors for dog attacks, koala deaths due to dog attacks comprised the cases and koala deaths due to vehicle collisions and chlamydia-like signs were the controls.

We used a Bayesian, mixed-effects logistic regression modelling approach to analyze these data^39^ where the probability of death for the *i*th koala ${\pi }_{i}$ was parameterized as a log-linear function of 1 to *m* risk factors:

1 $$log\frac{{\pi_{i} }}{{(1 - \pi_{i} )}} = \beta_{0} + \beta_{1} x_{1i} + \beta_{2} x_{2i} + \cdots + \beta_{m} x_{mi} + W(y_{i} ) + \varepsilon_{i}$$

In Eq. (1) ${\beta }_{0}$ represents the model intercept term and ${\beta }_{1} \cdots {\beta }_{m}$ the regression coefficients for each of the *m* explanatory variables included in the model. The term *y*_*i*_ denotes the location of death of the *i*th koala and *W*(*y*_*i*_) is a zero mean Gaussian process. The reason for including the *W*(*y*_*i*_) term in the model was to account for unexplained extra-binomial variation in cause-specific koala mortality risk arising from spatial autocorrelation in the data.

To facilitate inference, exceedance probability maps^40^ were developed to show the probability that the predicted proportion of koala deaths due to a given cause exceeded a given threshold (0.25 for dog attack mortalities and 0.75 for vehicle collision mortalities). Our choice of 0.25 and 0.75 for the exceedance probability maps was arbitrary and based primarily on the calculated proportional mortality risk for each cause of death. Our models were fitted using integrated nested Laplace approximation (INLA-SPDE^41^ available within the contributed R-INLA package^42^ in R.

## Results

Of the total number of reported deaths for the period 1 January 2009 to 31 December 2013 with location details, 395 were due to dog attacks, 1431 due to vehicle collisions and 943 due to chlamydia-like signs (Table 1).

The proportion of recorded koala deaths (proportional mortality risk) that were due to dog attacks by LGA ranged from 0.06 for Brisbane City to 0.22 for the Lockyer Valley (Table 2). LGAs with the highest proportions of recorded deaths due to dog attacks (in descending order) were Lockyer Valley (0.22, 95% CI 0.09–0.40), Logan (0.21, 95% CI 0.15–0.30), Toowoomba (0.19, 95% CI 0.08–0.36), Somerset (0.17, 95% CI 0.12–0.23) and Ipswich (0.16, 95% CI 0.09–0.25). The proportion of the study area with categories of registered dog densities (from ≤ 1 to > 50 dogs per square kilometer) is summarized in Supplementary Table S2 and a raster map of the estimated number of registered dogs per square kilometer is shown in Supplementary Fig. S1.

**Table 2:** Number of reported koala deaths due to dog attacks, total number of koala deaths due to all causes and proportional dog attack mortality risk, by Local Government Areas in South-East Queensland, Australia, 2009—2013.

Local government area	Number of koala deaths		Proportional Mortality risk (95% CI)
	Dog attacks	All causes	
Brisbane	11	184	6 (3–10)
Fraser Coast	4	53	8 (2–18)
Gold Coast	43	278	15 (11–20)
Gympie	1	12	8 (0–38)
Ipswich	15	93	16 (9–25)
Lockyer Valley	7	32	22 (9–40)
Logan	27	124	21 (15–30)
Moreton Bay	188	1207	16 (14–18)
Noosa	2	30	7 (1–22)
Redland	43	406	11 (8–14)
Scenic Rim	9	66	14 (6–24)
Somerset	33	191	17 (12–23)
South Burnett	2	27	7 (1–24)
Sunshine Coast	3	30	10 (2–27)
Toowoomba	7	36	19 (8–36)
Total	395	2769	14 (13–16)

**Figure 2:**
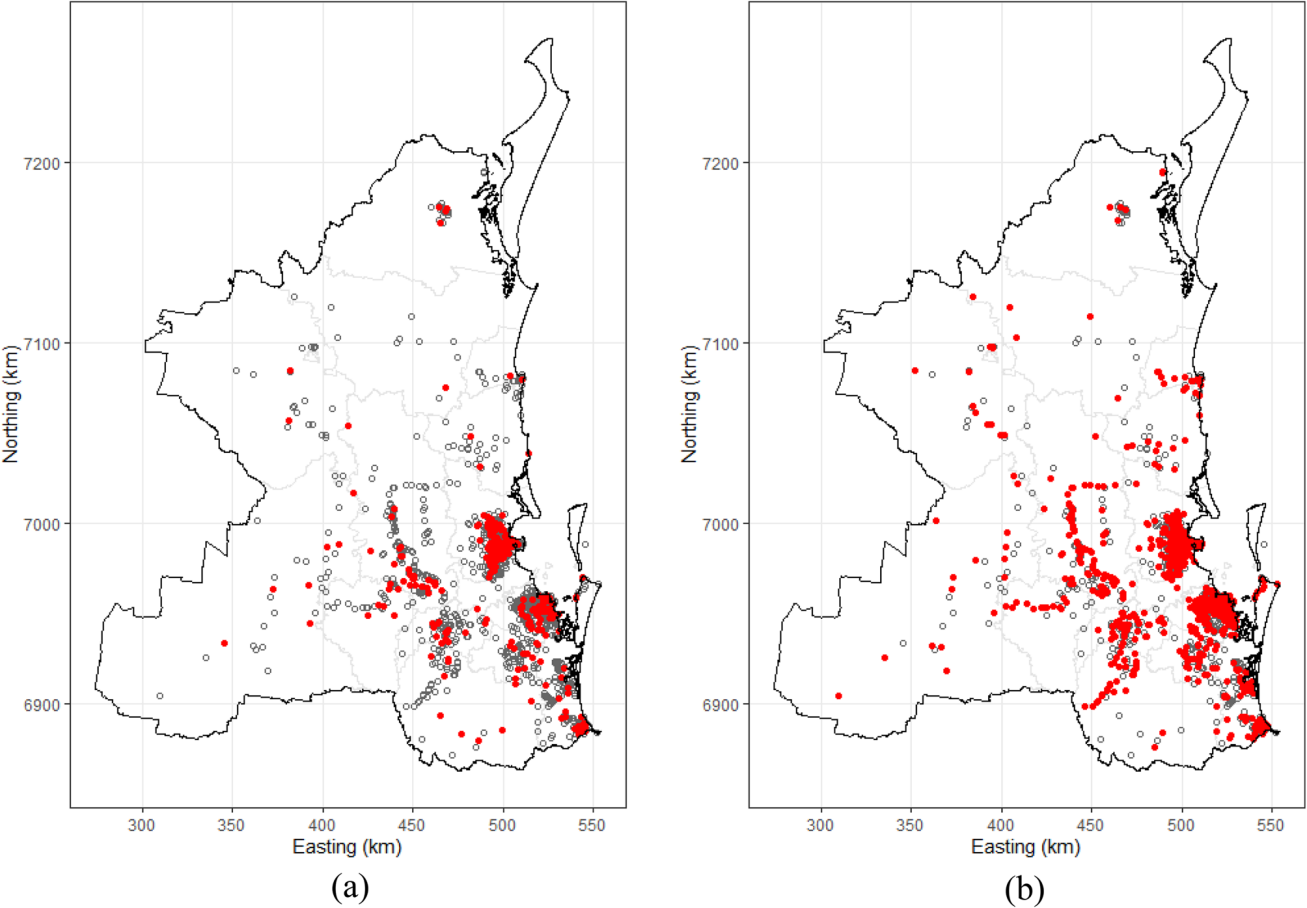
Point locations of koala deaths due to: (**a**) dog attacks (*n* = 395, red filled circles) and all other causes (*n* = 2374, white open circles); and (**b**) vehicle collisions (*n* = 1431, red filled circles) and all other causes (*n* = 1338, white open circles) in South-East Queensland, Australia, 2009–2013.

The proportion of recorded koala deaths that were due to vehicle collisions ranged from 0.11 for the Fraser Coast to 0.75 for Brisbane City (Table 3). LGAs with the highest proportions of recorded deaths due to vehicle collisions (in descending order) were Brisbane City (0.75, 95% CI 0.68–0.81), Scenic Rim (0.65, 95% CI 0.52–0.76), Noosa (0.63, 95% CI 0.44–0.80), Somerset (0.63, 95% CI 0.55–0.70) and South Burnett (0.59, 95% CI 0.39–0.78). In general, deaths due to vehicle collisions were more widely distributed across the study area compared with deaths due to dog attacks (Fig. 2a,b).

**Table 3:** Number of reported koala deaths due to vehicle collisions, total number of koala deaths due to all causes and proportional vehicle collision mortality risk, by Local Government Areas in South-East Queensland, Australia, 2009—2013.

Local government area	Number of koala deaths		Proportional Mortality risk (95% CI)
	Vehicle collisions	All causes	
Brisbane	138	184	75 (68–81)
Fraser Coast	6	53	11 (4–23)
Gold Coast	119	278	43 (37–49)
Gympie	5	12	42 (15–72)
Ipswich	50	93	54 (43–64)
Lockyer Valley	16	32	50 (32–68)
Logan	60	124	48 (39–58)
Moreton Bay	622	1207	52 (49–54)
Noosa	19	30	63 (44–80)
Redland	184	406	45 (40–50)
Scenic Rim	43	66	65 (52–76)
Somerset	120	191	63 (56–70)
South Burnett	16	27	59 (39–78)
Sunshine Coast	14	30	47 (28–66)
Toowoomba	19	36	53 (35–70)
Total	1431	2769	51 (50–53)

Estimated regression coefficients and their standard errors from the mixed-effects logistic regression model of factors associated with the odds of death due to dog attack are shown in Table 4. Koala deaths due to dog attack were positively associated with registered dog population density and negatively associated with lot density. Koala deaths due to vehicle collisions were positively associated with road density (road length per square kilometer) and negatively associated with human population density and distance from primary and secondary roads (Table 5). The variance of the spatial random effect term for the vehicle collision model was greater than the variance of the spatial random effect term for the dog attack model (Tables 4 and 5) indicative of greater variability in the amount of spatial dependence in the vehicle collision mortality locations. In practical terms this means that there were some locations in the study area with aggregations of koala deaths due to vehicle collisions unaccounted-for by human population density, elevation, distance to primary and secondary roads and road density, and indicative of area-specific (i.e., local) road-related hazards.

**Table 4:** Cause-specific risk factors for koala mortalities due to dog attacks in South-East Queensland, Australia, 2009–2013. Estimated regression coefficients and their standard errors were derived from a mixed-effects logistic regression model of risk factors associated with the odds of death due to dog attacks.

Explanatory variable	Coefficient	SD	Odds ratio (95% CrI)
Intercept	− 1.9319	0.2241	
Human population density (× 1000)^a^	0.3572	0.2974	1.43 (0.80–2.56)
Median elevation (× 100 m)^b^	0.0122	0.0893	1.01 (0.84–1.20)
Dog population density (× 10)^c^	0.0348	0.0158	1.04 (1.00–1.07)
Remoteness (× 60 min)^d^	− 0.0001	0.0020	1.00 (1.00–1.00)
Lot density (× 10)^e^	− 0.0376	0.0126	0.96 (0.94–0.99)

Spatial random effect:	Mean	SD	95% CrI
Variance	0.7641	0.4978	0.0919–1.736
Smoothness, $\kappa$	0.0002	0.0002	0–0.0006
Range, $\phi$, (km)	17.5	13.7	1.98–43.8

*SD* standard deviation, *CrI* credible interval.

^a^Number of individuals per square kilometer (1000 individual increments).

^b^Height above sea level (100 m increments).

^c^Number of dogs per square kilometer (10 dog increments per square kilometer).

^d^Time taken to reach a city of more than 50,000 population (60 min increments).

^e^Number of lots per square kilometer (10 lot increments per square kilometer).

**Table 5:** Cause-specific risk factors for koala mortalities due to vehicle collisions in South-East Queensland, Australia, 2009–2013. Estimated regression coefficients and their standard errors were derived from a mixed-effects logistic regression model of risk factors associated with the odds of death due to vehicle collision.

Explanatory variable	Coefficient	SD	Odds ratio (95% CrI)
Intercept	0.2943	0.2533	
Human population density (× 1000)^a^	− 0.2095	0.0710	0.81 (0.71–0.93)
Median elevation (× 100)^b^	− 0.0134	0.0865	0.99 (0.83–1.17)
Distance to primary road (km)	− 0.0339	0.0177	0.97 (0.93–1.00)
Distance to secondary road (km)	− 0.0014	0.0232	1.00 (0.95–1.04)
Road density (× 1000)^c^	0.0519	0.0096	1.05 (1.03–1.07)

Spatial random effect	Mean	SD	95% CrI
Variance	6.9672	5.6463	1.240–17.60
Smoothness, $\kappa$	0.0004	0.0002	0.0002–0.0009
Range, $\phi$, (km)	7.5	2.9	2.1–13.1

*SD* standard deviation, *CrI* credible interval.

^a^Number of individuals per square kilometer (1000 individual increments).

^b^Height above sea level (100 m increments).

^c^Road density per square kilometer (1000 m increments in road length per square kilometer).

**Figure 3:**
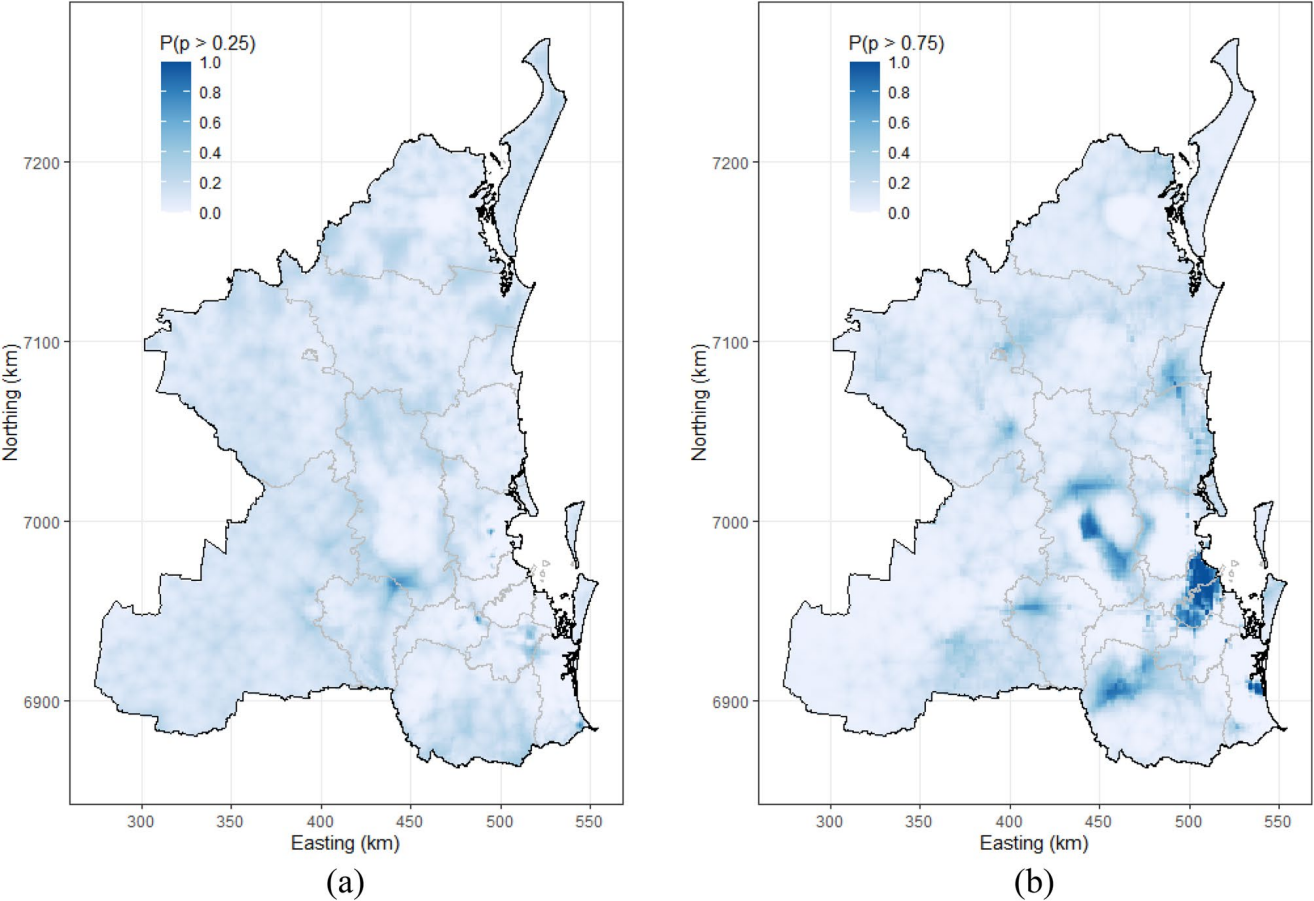
Raster maps showing the geographic distribution of the probability that the proportion of koala deaths due to: (**a**) dog attack exceeded 25% (0.25); and (**b**) vehicle collision exceeded 75% (0.75) of all koala deaths in the study area of South-East Queensland, Australia, 2009–2013. The exceedance probabilities for these plots were derived from the Bayesian, mixed-effects logistic regression models presented in Tables 4 and 5.

Raster maps showing the predicted proportion of koala deaths due to dog attacks and vehicle collisions are shown in Supplementary Fig. S2a,b. Exceedance probability maps for koala deaths due to dog attack and vehicle collision are shown in Fig. 3a,b, respectively. Care is required with interpretation here. Figure 3a,b do not show the probability of death for a given cause, rather the probability of dog attacks accounted for more than 25% of all koala deaths across the study area for 2009–2013 (Fig. 3a) and the probability of vehicle collisions accounted for more than 75% of all koala deaths across the study area for 2009–2013 (Fig. 3b).

**Figure 4:**
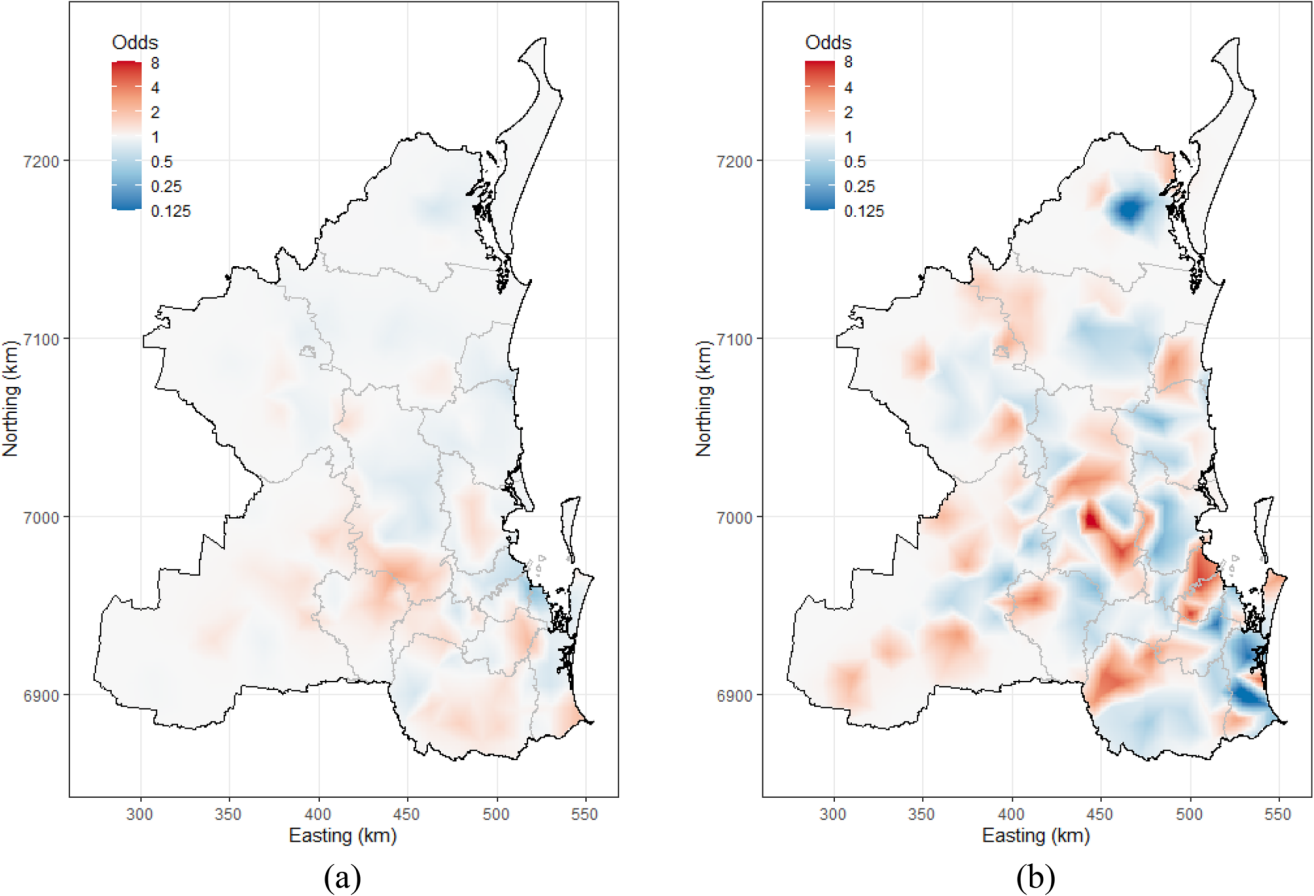
Raster maps showing the geographic distribution of the relative increase or decrease in the spatially correlated, unaccounted-for odds of mortality risk due to (**a**) dog attacks and (**b**) vehicle collisions for one unit increases in the spatial random effect term for each model, South-East Queensland, Australia, 2009–2013. The spatially correlated unaccounted-for odds for these plots were derived from the Bayesian, mixed-effects logistic regression models presented in Tables 4 and 5.

Figure 4a,b show the relative increase or decrease in the spatially correlated, unaccounted-for odds of mortality due to dog attacks (Fig. 4a) and vehicle collisions (Fig. 4b) for one unit increases in the spatial random effect term for each model. We use the term “risk” as our descriptor for this measure. Compared with the vehicle collision model the spatially correlated, unaccounted-for mortality risk for dog attack were relatively homogenous across the study area with a focus of elevated risk centered around the north of Lockyer Valley and the south of Somerset LGAs (Fig. 4a). Unaccounted-for mortality risk for vehicle collisions were more variable with the dark red shaded areas in Fig. 4b identifying locations where there was a relatively high proportion of recorded koala deaths attributable to vehicle collision.

## Discussion

Previous research has highlighted that urban sprawl has contributed to koala population declines by fragmentation of koala habitat through the expansion of road networks and human settlements^20,43^. In this study we quantified the association between anthropogenic and environmental risk factors and the two main causes of koala deaths: dog attacks and vehicle collisions.

We identified a moderately increased risk of koala deaths due to dog attack in areas of higher dog population densities. High risk areas for deaths due to dog attacks were located in the south-western part of the study area: the north of the Lockyer Valley and the south of Somerset LGAs (Supplementary Fig. S2a, Figs. 3a and 4a) and targeted investigations into the reasons for this elevated risk would be a logical extension to the analyses presented in this study. Koala deaths due to dog attacks occur mainly in the weak, young^44^ or sick animals^12^. Being a slow-moving animal on the ground, koalas may become an easy target for dogs in areas where koala habitat is fragmented, in particular during the breeding season when male koalas move frequently in search of female partners^45^. In urban areas koalas are mainly attacked by domestic dogs, while attacks in bushland or forests are often due to free-roaming (wild) dogs^46,47^. Strict regulations on the management of domestic dogs combined with awareness campaigns to actively promote responsible dog ownership need to be applied to reduce domestic dog koala attacks^48^. Our map of the distribution of dogs across the SEQLD study area (Supplementary Fig. S1) was prepared using registered dog population data obtained from local councils. Registration of dogs with councils is mandatory in Australia and perhaps more strictly adhered to in urban compared with rural areas. Nevertheless, information campaigns, such as “Koalas and Dogs Don’t Mix”^49^ that raise public awareness of the potential hazards that dogs can inflict on koalas and remind the public of the need to leash dogs or keep them in fenced areas, particularly in koala dense areas, are highly recommended.

The exceedance probability map shown in Fig. 3a should be interpreted with caution as details concerning the types of dog involved in the attack events were not routinely recorded. Since elevated free-roaming or wild dog densities are likely to be associated with a higher risk for koala mortality compared with registered dog density, it would have been ideal to use free-roaming dog density as an additional explanatory variable in our analyses, but these data were not available. Wild dogs and dingoes are known predators of koalas^50,51^ and future investigations of koala deaths due to canine attacks should consider using estimates of the density of free-roaming dogs (including dingoes) as candidate risk factors for modelling. This would enhance the detail of recommendations derived from koala mortality risk models by distinguishing between risk factors for deaths due to free-roaming canines and registered dogs. Fencing of important koala habitats and application of measures to control free-roaming dog numbers can limit encounters between koalas and dogs, decreasing dog attack mortality risk^1^.

We identified a moderately decreased risk of koala deaths due to vehicle collision with increased distance to primary and secondary roads. It was expected to identify such an association between vehicle collision mortality risk and distance to roads, since koalas—if they were to die from a vehicle collision, they would do so in relatively close proximity to the site where the collision occurred. Assuming a relatively constant surveillance effort across the 5-year study period, a useful finding from these analyses is the identification of locations where there was an excess of vehicle collision mortality risk, after known risk factors had been accounted for (Fig. 4b). This identifies specific areas (i.e., the red shaded areas in Fig. 4b) where intervention efforts need to be applied. Building new roads often fragments existing wildlife habitat and influences movement behavior of koalas as koalas are dependent on specific habitat areas and tree species for food. It needs to be noted that koala vehicle collision risk is not only dependent on the presence of road networks, but also on speed limits, vehicle types and traffic volumes^20,44,52^. Highways, where vehicles travel at relatively high speeds, result in more fatal injuries compared to roads where speeds are substantially lower^20^. Measures have been implemented to reduce the risk of koala vehicle collisions^20^, for example erection of road signs to alert motorists to reduce their speed in koala habitat areas^53^. Over- and underpasses or culverts under roads have also been deployed to ensure safe movement of koalas^54^. Interestingly, the risk for vehicle collisions with koalas was found to be elevated in some less densely populated areas (Fig. 3b), as perhaps less attention has been placed on protecting koalas from vehicle collisions in these more remote locations^48^.

Because our analyses were based on a subset of all koala deaths that occurred during the defined study period, we were unable to provide estimates of absolute mortality risk. Instead, we report, for koalas presented to the participant wildlife hospital, the relative proportions of koala deaths due to specific causes (Tables 2 and 3). For our regression analyses, we used a case–control approach with appropriate inference based on the assumption of non-differential misclassification^55^. In the context of this study we have assumed that the probability of: (a) reporting a koala death; and (b) correctly classifying the cause for a koala death was similar for dog attacks and vehicle accidents. Non-differential misclassification results in point estimates of risk being biased towards the null, which could result in some risk factors might being dismissed due to a statistically non-significant association with the reason of death.

The models described in this study were developed using explanatory variables presented in raster maps with cell dimensions of 500 m by 500 m. It could be argued that this might not have been the best resolution for some variables, as there might have been substantially greater spatial variability at finer spatial scales. It also needs to be acknowledged that the explanatory variables included in the models were restricted to publicly available spatial data sets and, as a result, were likely to be crude predictors of koala mortality risk. Thus, there is a trade-off when analyzing koala mortality data: either (a) elect to use data for a relatively large study area, necessitating the use of relatively crude explanatory variables where the bias in the measurement of those variables is likely to be relatively uniform; or (b) analyze data for a smaller study area where greater effort can be expended to collect more detailed explanatory variable data. For this study we elected to use the former approach, driven by a need to provide estimations of koala mortality risk across relatively large areas of SEQLD koala habitat.

A limitation of this study, and in fact of any research on koala populations, is a potential underreporting of koala mortalities. In contrast, there might be resident sentiments in certain areas with strong koala awareness campaigns that impact behaviors in observing koalas, which results in overreporting of koalas. Also, there might be fluctuation in koala population densities across years^56^. To address these issues our analysis used koala mortality data reported over a 5-year time period. The variables used in the models were reasoned to be biologically plausible risk factors for koala mortalities. It could be argued that characteristics of some risk factors used in the models might have changed over time due to infrastructural improvements, community efforts, and environmental challenges (i.e., floods and bush fires). However, the relative magnitude of the strength of the association between each of our hypothesised risk factors as determinants of koala mortality risk is reasoned to be relatively invariant over the period of time between data collection and inference (10 years in the case of this study).

In conclusion, this study provides useful insights into the spatial distribution of preventable koala deaths in SEQLD, and factors associated with these deaths. The use of citizen science data allowed for a comprehensive analysis of the relationship between anthropogenic factors and the leading causes of koala mortality. The results highlight the importance of considering the risk of both dog attacks and vehicle collisions in the development of conservation strategies to protect koala populations. Future research could build on this study by incorporating estimates of free-roaming dog and dingo population densities. Ultimately, the findings of this study can inform targeted conservation efforts to reduce preventable koala deaths and promote the long-term survival of this iconic endangered animal species.

### Supplementary Information

Supplementary Information.

## Data Availability

The datasets used and analyzed during the current study are available from the corresponding author on reasonable request.
