# Supplementary material for: Anthropogenic and environmental factors associated with koala deaths due to dog attacks and vehicle collisions in South-East Queensland, Australia, 2009–2013
Source: Sci Rep. 2023 Aug 31;13:14275. doi: 10.1038/s41598-023-40827-w (PMC10471613; doi:10.1038/s41598-023-40827-w)
Supplement: Supplementary file 1 — Supplementary Information. [file 41598_2023_40827_MOESM1_ESM.docx]

**Supplementary Table S1**: Number of registered dogs per Local Government Area in South-East Queensland, Australia, 2018.

| **Local government area** | **Number of SALs** | **Number of dogs** |
| --- | --- | --- |
| Brisbane | 193 | 105,603 |
| Fraser Coast | 97 | 18,401 |
| Gold Coast | 79 | 64,375 |
| Gympie | 104 | 7503 |
| Ipswich | 79 | 32,623 |
| Lockyer Valley | 76 | 7099 |
| Logan | 66 | 40,643 |
| Moreton Bay | 95 | 68,565 |
| Noosa | 24 | 8915 |
| Redland | 23 | 26,605 |
| Scenic Rim | 113 | 5272 |
| Somerset | 73 | 4112 |
| South Burnett | 101 | 5526 |
| Sunshine Coast | 118 | 41,236 |
| Toowoomba | 219 | 19,762 |
| **Total** | **1461** | **456,240** |

**Supplementary Table S2**: Area (in square kilometers) as a function of categorized registered dog densities, South-East Queensland, Australia, 2018. The numbers in parentheses show the percentage of the total study area in each registered dog density range.

| **Number of dogs per km^2^** | **Area km^2^ (%)** |
| --- | --- |
| 0 to ≤1 | 40,481 (70) |
| 1 to ≤2 | 3,070 (5) |
| 2 to ≤10 | 5,962 (10) |
| 10 to ≤20 | 1,911 (3) |
| 20 to 50 | 2,176 (4) |
| >50 | 4,024 (7) |
| **Total** | **57,623 (100)** |

| 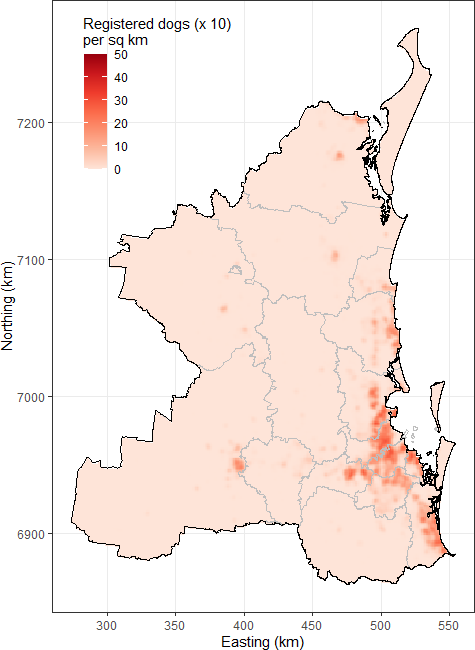 |
| --- |
| **Supplementary Figure S1:** Raster map showing the estimated number of registered dogs per square kilometer in South-East Queensland, Australia, 2018. The map boundaries for this (and subsequent maps in the supplementary material for this paper) were obtained from the Australian Bureau of Statistics (URL: https://www.abs.gov.au/). This map (a subsequent maps in the supplementary material for this paper) were created using R using the contributed sf, raster and ggplot2 packages. |

| 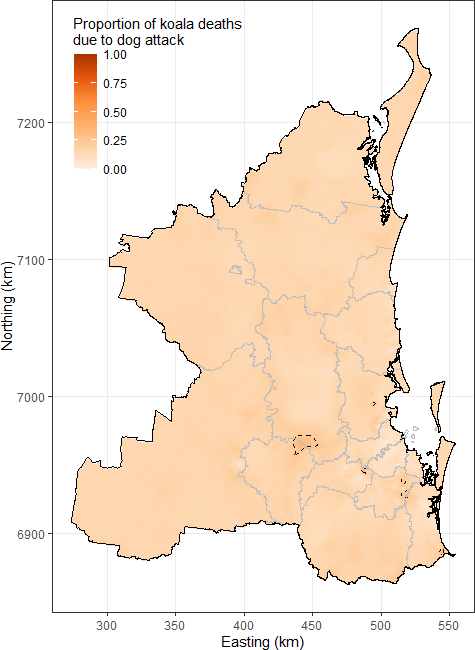 | 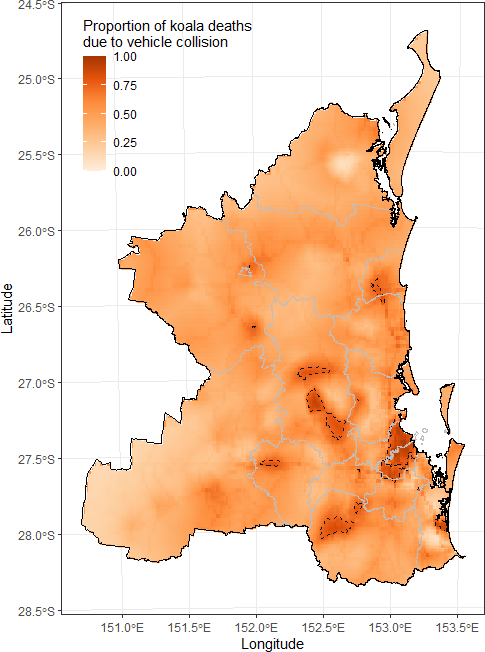 |
| --- | --- |
| (a) | (b) |
| **Supplementary Figure S2**: Raster maps showing the predicted proportion of koala deaths due to: (a) dog attacks; and (b) vehicle collisions in South-East Queensland, Australia, 2009-2013. In (a) the dashed contour lines delineate areas where the predicted proportion of deaths due to dog attacks exceeded 0.25. In (b) the dashed contour lines delineate areas where the predicted proportion of deaths due to vehicle collisions exceeded 0.75. The predicted proportions of koala deaths shown in these plots were derived from the Bayesian, mixed-effects logistic regression models presented in Tables 4 and 5. | |
